# Anti-PECAM-1 antibodies: another tool for visualization of Reissner´s fiber and the subcommissural organ in rat central nervous system

**DOI:** 10.1007/s00429-026-03088-7

**Published:** 2026-04-04

**Authors:** Lukáš Malčický, Ján Košuth, Daniel Barčák, Jarmila Zrubáková, Marie Vancová, Zuzana Daxnerová, Juraj Ševc, Anna Alexovič Matiašová

**Affiliations:** 1 Department of Cell Biology, Institute of Biology and Ecology, Faculty of Science, Pavol Jozef Šafárik University in Košice, Košice, Slovak Republic; 2 Institute of Parasitology, Slovak Academy of Sciences, Košice, Slovak Republic; 3 Laboratory of Electron Microscopy, Institute of Parasitology, Biology Centre, Czech Academy of Sciences, České Budějovice, Czech Republic

**Keywords:** PECAM-1 (CD31), Reissner´s fiber, Subcommissural organ, Brain ventricular system, Rats

## Abstract

Reissner´s fiber (RF) is an extracellular structure formed by the condensation of material released by cells of the subcommissural organ (SCO). RF and SCO are involved in the morphogenesis of the central nervous system (CNS) in various species, from lower vertebrates to mammals. Visualization of RF and the SCO may be challenging due to the absence of markers and the limited number of commercially available antibodies (e.g., anti-SCO-spondin). In this work we present that anti-platelet endothelial cell adhesion molecule-1 (anti-PECAM-1) antibodies, which recognize the extracellular region of PECAM-1, bind to the RF and to the apical surface of SCO cells in rat CNS. According to our analyses, PECAM-1 represents previously unrecognized component of the RF, which is not produced by the SCO cells, but rather is incorporated into RF via SCO cells through as-yet-unknown mechanisms.

**Supplementary Information:**

The online version contains supplementary material available at 10.1007/s00429-026-03088-7.

## Introduction

The subcommissural organ (SCO) of vertebrates belongs to a group of circumventricular organs with the secretory functions located within the brain ventricular system. The SCO is formed by a specialized population of ependymal cells lining the dorso-caudal region of the third ventricle beneath the posterior commissure, just above the opening to the cerebral aqueduct (Duvernoy and Risold 2007; Gobron et al. 1999; Muñoz et al. 2019; Rodríguez et al. 1998). The unique ultrastructure of the cells of the SCO facilitates the production of high-molecular-mass glycoproteins released via secretory granules into the cerebrospinal fluid (CSF) (Chen et al. 1973; Nualart et al. 1991). The main known secretory product of the SCO is SCO-spondin, representing a matricellular protein belonging to the thrombospondin superfamily (Gobron et al. 1996, 2000). SCO-spondin, encoded by the *Sspo* gene (Gonçalves-Mendes et al. 2003; Meiniel et al. 1995; Popescu et al. 1997), is a multidomain protein with a modular organization and numerous potential N-glycosylation sites. SCO-spondin consists of several thrombospondin-type I repeat (TSR) domains, low density lipoprotein (LDL) receptor-type A repeats, epidermal growth factor (EGF)-like domains, and von Willebrand factor (vWF) domains, as identified in the central nervous system (CNS) of bovines (Gobron et al. 1996, 2000; Meiniel et al. 2008; for a review, see Meiniel and Meiniel 2007, Sepúlveda et al. 2021). SCO-spondin aggregates on the apical surface of SCO cells, forming pre-Reissner’s fiber (pre-RF). Subsequently, this material is organized into a long, thread-like structure termed Reissner´s fiber (RF), first identified in the nervous system of lampreys (Reissner 1860). RF passes through the cerebral aqueduct, the fourth brain ventricle, the central canal (CC) of the spinal cord, and finally disintegrates in its terminal region, forming the *massa caudalis*, which represents glycoproteins, presumably released from the CNS via nearby vasculature (Molina et al. 2001; Rodríguez et al. 1987, 1998).

The SCO and RF have been studied in various vertebrate species, including fishes (Bellegarda et al. 2023; Cantaut-Belarif et al. 2018; Tian et al. 2025; Troutwine et al. 2020), amphibians (Diederen 1970), reptiles (Fernández-Llebrez et al. 1987; Peruzzo et al. 1990), birds (Didier et al. 1992; Stanic et al. 2010), and mammals (Castenholz and Zöltzer 1980; Muñoz et al. 2019; Vio et al. 2000), particularly bovine (Meiniel et al. 1990, 1995; Muñoz et al. 2019; Pérez et al. 1996) and rodents (Corales et al. 2022; Muñoz et al. 2019; Nualart et al. 2023; Vio et al. 2008; Xu et al. 2023). In human CNS, the SCO is established early in the embryonic period. Interestingly, its products remain in a soluble form, without aggregating into the RF (Rodríguez et al. 1992, 2001), a phenomenon similar to that reported in other mammalian species, such as bats, camels, and chimpanzees (Galarza 2002).

SCO‒RF development and function have been studied in embryonic and larval stages using genetic models, GFP-targeting techniques, and imaging after injection of specific tracers (Bellegarda et al. 2023; Cantaut-Belarif et al. 2020; Lu et al. 2020; Tian et al. 2025; Xu et al. 2023; Zhang et al. 2024). Mutations of the *sspo* gene in zebrafish are linked to the development of idiopathic-like scoliosis and neuroinflammation (Rose et al. 2020). Developmental deformities have also been reported in *Sspo* knockout mice, including small brain ventricles lacking RF material and minor defects in spine geometry (Xu et al. 2023). Although the function of the SCO‒RF complex is not clear, the early establishment of the SCO in the embryonic period (for review see Grondona et al. 2012; Meiniel et al. 1990; Schoebitz et al. 1986) indicates a significant role of SCO cells and their products in CNS development, such as promotion of neurite growth (Gobron et al. 2000; Hoyo-Becerra et al. 2010; Meiniel 2001) and a role in cortical development. This was documented in a mouse model with genetic ablation of SCO cells, resulting in a gradual loss of cortical neurons and the development of hydrocephalus (Zhang et al. 2024). The SCO and/or RF can be visualized by immunolabeling using anti-SCO-spondin antibodies (Corales et al. 2022) and antisera raised against bovine RF (termed AFRU) (Muñoz et al. 2019; Rodríguez et al. 1984). Another documented molecule represents galectin-1 (Gal-1), which binds the dimers of SCO-spondin and secures its oligomerization (Muñoz et al. 2019). Additionally, due to the adhesive function of RF, catecholamines and serotonin present on its surface may be detected (Caprile et al. 2003).

In this study, we report that antibodies raised against platelet endothelial cell adhesion molecule-1 (PECAM-1, CD31) may be applied for visualization of RF and apical surface of SCO cells in the CNS of rats. PECAM-1 is an integral type I transmembrane glycoprotein, typically present in cells of the vascular system. It is expressed on the surface of blood cells including T and B cells, monocytes, granulocytes or platelets and on the vascular endothelial cells (Newman et al. 1990; Simmons et al. 1990). The membrane-bound protein is capable of cell-cell homophylic or heterophylic interactions, which may be influenced by alternative splicing (Yan et al. 1995, Sun et al. 1996). The protein is distributed over the entire surface of endothelial cells, in specialized cell-cell junctions (Simmons et al. 1990; Feng et al. 2004) to maintain vascular permeability barrier. The PECAM-1 plays an important role in transendothelial migration of leukocytes during neuroinflammation (reviewed in Kalinowska and Losy 2006). At pathological conditions or during apoptosis, extracellular portion of PECAM-1 may be enzymatically cleaved from the surface of endothelial or T cells to form the soluble circulating sPECAM-1, present in serum or CSF (Fornasa et al. 2010; Ilan et al. 2001; Magnoni et al. 2025; Tran-Dinh et al. 2022; Zaremba et al. 2002). In this work, we demonstrate that anti-PECAM-1 antibodies are applicable in immunolabelling of RF and SCO cells using confocal and electron microscopy. In addition, we analyzed SCO cells using laser capture microdissection and RT-qPCR. Interestingly, our data indicate that the target of anti-PECAM-1 antibodies, PECAM-1, present in RF and apical cytoplasm of SCO cells, is not of SCO origin, but rather represents an external component, potentially incorporated into RF by SCO cells through unknown mechanisms.

## Materials and methods

### Experimental animals

All experiments were performed in accordance with the ARRIVE guidelines, and approval of the National Veterinary and Food Administration of the Slovak Republic and the Animal Care Committee of P. J. Šafárik University in Košice, Slovakia, in accordance with the European Communities Council Directive 2010/63/EU and in compliance with current national legislation under no. Ro-1455/18–221/3 and Ro-3051-5/2021 − 220. Wistar albino rats were obtained from Velaz (Prague, Czech Republic). Animals were housed under standard laboratory conditions with a 12-h light/12-h dark cycle. Each animal was fed a complete and balanced standard laboratory diet for rats and mice (Altromin) and had *ad libitum* access to food and water. Analyses were performed on the tissues isolated from the intact brain and spinal cord of Wistar albino rats at the age of 8 postnatal days (P8, neonatal), 29 days (P29, preadolescent), 90 days (P90, young adult), and 18 months (M18, senescence).

### Tissue isolation

Animals were terminally anaesthetized by intraperitoneal (*i.p.*) overdose of sodium thiopental and perfused with heparinized saline followed by fixation solution: freshly prepared 4% paraformaldehyde (PFA, Sigma-Aldrich, St. Louis, MO, USA, #158127) in 0.1 M phosphate buffer (PB) at pH 7.4 (for immunofluorescence, *n* = 3, for each studied time point); 2.5% glutaraldehyde (GA, Sigma-Aldrich, #G5882) and 2% PFA (for ultrastructure, *n* = 3, P29); or 4% PFA and 0.1% GA (for immunogold labeling, *n* = 3, P29). For scanning electron microscopy (SEM) analyses (*n* = 3, M18) or gene expression analyses (*n* = 3, P90), animals were perfused with heparinized saline only. After isolation, tissue for gene expression analyses was rapidly frozen at −80 °C and processed according to the Laser Capture Microdissection (LCM) protocol (Garcia-Ovejero et al. 2018). For each animal, 5–10 sections were used per analysis (depending on the purpose and thickness of the samples).

### Ultrastructural analysis

#### Transmission electron microscopy (TEM)

Isolated samples of the brain and the lumbar region of the spinal cord were post-fixed in 2.5% GA and 2% PFA overnight, then transferred to PB and cut to 200 μm-thick coronal sections using a vibratome, Leica VT1200S (Leica Microsystems, Mannheim, Germany). Tissue sections were washed in PB and post-fixed/contrasted in 2% OsO_4_ (EMS, Hatfield, PA, USA #19150) for 2 h at room temperature. After the washing step, tissue sections were dehydrated in increasing grades of ethanol (EtOH) (30% EtOH < 50% EtOH < 70% EtOH < 96% EtOH < 100% EtOH for 15 min each) and infiltrated by a mixture of epoxy resin Epon (Serva Electrophoresis, Heidelberg, Germany, prepared according to Hayat 1972) and EtOH (1:3, 1:1, 3:1, respectively, for 1 h each). Next, the samples were infiltrated with epoxy resin for 24 h, transferred to fresh resin, and left to polymerize for an additional 48 h at 60 °C. Embedded samples were mounted on epoxy resin capsules, then trimmed and cut to 200 nm-thick sections using a Leica EM UC7 ultramicrotome (Leica Microsystems) for the identification of the SCO in the brain sections and RF in the CC of the spinal cord using 0.1% Toluidine Blue staining. Afterward, samples were cut to 70 nm-thick ultrathin sections, transferred to formvar-coated slot or mesh copper grids, contrasted in 2% aqueous uranyl acetate followed by lead citrate, washed, dried, and analyzed with a JEM-1230 transmission electron microscope (JEOL, Tokyo, Japan).

#### Scanning electron microscopy

The apical surface of cells lining the brain ventricular system (third ventricle, cerebral aqueduct, fourth ventricle) or the CC of the spinal cord was exposed by a medial cut. Isolated tissues were fixed by immersion in a mixture of 2.5% GA and 2% PFA in PB (pH 7.4) for 1 h. Fixed samples were washed in 0.1 M PB, dehydrated in increasing grades of EtOH (30% EtOH for 5 min, 50% EtOH for 5 min, 70% EtOH for 2 × 10 min, 96% EtOH for 3 × 10 min, and 100% EtOH for 3 × 10 min). After dehydration, samples were immersed in 1,1,1,3,3,3-Hexamethyldisilazane (Merck Millipore, Darmstadt, Germany, #8.04324), air-dried, and mounted on aluminum pins. Before analysis using the scanning electron microscope JSM-6510 LA (JEOL), samples were coated with a thin layer of gold using a sputter coater JFC-1300 (JEOL).

### Immunofluorescent labeling

Brains and spinal cords were cut into 40 μm-thick coronal or longitudinal sections using a Leica CM1860 Cryostat (Leica Biosystems, IL, USA). Tissue sections were washed with 0.1 M phosphate buffer saline (PBS) at pH 7.4, and non-specific protein activity was blocked using 5% ChemiBLOCKER™ (Merck Millipore, Burlington, MA, USA, #2170) diluted in PBS with 0.3% Triton X-100 for 30 min. Samples were incubated with primary antibodies (Table 1) diluted in PBS with 1% ChemiBLOCKER and 0.3% Triton X-100 at 4 °C overnight. Control tissues with omitted primary antibodies were also prepared. Sections were washed in PBS and incubated with secondary antibodies (Table 2) diluted in PBS with 1% ChemiBLOCKER and 0.3% Triton X-100 at room temperature in the dark for 2 h. After incubation, tissue sections were washed in PBS. To visualize cell nuclei, samples were counterstained with DRAQ5^®^ (1:500, Cell Signaling Technology, Leiden, Netherlands, #4084) for 10 min, washed in PBS, transferred to glass slides and cover-slipped using ProLong Gold Antifade Reagent with DAPI (Cell Signaling Technology, #8961). Samples were analyzed using the confocal system Leica TCS SP5 X equipped with LAS AF software (Leica Microsystems) using x40, and x100 objective lens and WLL or argon laser. To capture the images, XYZ mode was used (resolution 8 bits, 1024 × 1024 pixels or 2048 × 2048 pixels, scanning speed 100 Hz, gain 600–750 V). To visualize the fluorophores, following Ex λ and Em λ were used: AF488 (Ex 488 nm, Em 500–540 nm), AF555 (Ex 555 nm, EM 565–590 nm), AF594 (Ex 594 nm, EM 610-635 nm), and DRAQ5 (Ex 647 nm, EM 657–690 nm).

### Post-embedding immunogold labeling

Isolated samples of the brain and lumbar region of the spinal cord were post-fixed in 4% PFA and 0.1% GA overnight, transferred to PB, and cut to 200 μm-thick coronal sections using a vibratome, Leica VT1200S (Leica Microsystems). Tissue sections were dehydrated in increasing grades of EtOH (30% EtOH < 50% EtOH < 70% EtOH < 96% EtOH < 100% EtOH for 15 min each) and infiltrated with a mixture of LR White embedding resin medium grade (LRW, EMS, #14381) and EtOH (1:3, 1:1, 3:1, respectively, for 1 h each). Afterward, the samples were infiltrated with LRW for 24 h, transferred to fresh LRW, and left to polymerize for an additional 48 h at 50 °C. After polymerization, semi-thin sections were cut and stained with 0.1% Toluidine Blue to identify the SCO and CC region in the spinal cord. Ultrathin sections were transferred to mesh nickel grids, incubated in 3% BSA-c™ (Aurion, Wageningen, Netherlands, #900.009) in 0.1 M HEPES and Tween 20 to block non-specific protein activity for 1 h, followed by incubation with primary antibodies (Table 1) for another 1 h. Next, samples were washed in 3% BSA-c in 0.1 M HEPES and Tween 20 and incubated with secondary antibodies (Table 2) diluted in 3% BSA-c in 0.1 M HEPES and Tween 20 for 1 h. After incubation, the samples were washed in 0.5% BSA-c in 0.1 M HEPES, followed by washing in dH_2_O, and then contrasted using 2% aqueous uranyl acetate for 5 min. Afterward, samples were washed, dried, and analyzed by JEM 1010 (JEOL) and JEM-1230 (JEOL) transmission electron microscope.

**Table 1:** List of primary antibodies used in analyses

Primary antibody	Host species/clonality	Immunogen	Manufacturer	Cat. number	RRID	Dilution
Anti-human/mouse/rat CD31/PECAM-1	Goat polyclonal	Mouse CD31/PECAM-1 aa 18–590 (Q08481), recombinant (extracellular region)	R&D systems/bio-techne	AF3628	AB_2161028	1:500 (IF) 1:20 (IEM)
Anti-CD31 [EPR17260-263]	Rabbit monoclonal	Mouse CD31 aa 400–600 (Q08481), recombinant (extracellular region)	abcam	ab222783	AB_2905525	1:100 (IF)
Anti-CD31/PECAM-1	Rabbit polyclonal	Human CD31 aa 700–738 (P16284) (intracellular region)	NOVUS BIOLOGICALS	NB100-2284	AB_10002513	1:100 (IF)
CD31 (PECAM-1) monoclonal (clone 390)	Rat monoclonal	N/A, reacts with mouse CD31, applicable also for rats and other species (region not specified)	Thermo fisher scientific - invitrogen -eBioscience™	14–0311-85	AB_467202	1:100 (IF)
Anti-PAX6 [EPR15858]	Rabbit monoclonal	N/A (proprietary)	abcam	ab195045	AB_2750924	1:350 (IF)
Anti-SSPO	Rabbit polyclonal	Synthetic peptide derived from human SCO-spondin (SSPO)	Biorbyt	orb507583	NR	1:300 (IF)
Anti-Vimentin	Chicken polyclonal	Human full length Vimentin (P08670), recombinant	abcam	ab24525	AB_778824	1:500 (IF)

**Table 2:** List of secondary antibodies used in analyses

Secondary antibody	Manufacturer	Cat. number	RRID	Dilution
donkey anti-goat IgG H&L (Alexa Fluor^®^ 488)	abcam	ab150129	AB_2687506	1:500 (IF)
Alexa Fluor^®^ 594 AffiniPure^®^ donkey anti-goat IgG (H + L)	Jackson ImmunoResearch	705-585-003	AB_2340432	1:500 (IF)
donkey anti-rabbit IgG H&L (Alexa Fluor^®^ 488)	abcam	ab150073	AB_2636877	1:500 (IF)
donkey anti-rabbit IgG H&L (Alexa Fluor^®^555)	abcam	ab150074	AB_2636997	1:500 (IF)
Alexa Fluor^®^594 AffiniPure^®^ donkey anti-rat IgG (H + L)	Jackson ImmunoResearch	712-585-153	AB_2340689	1:500 (IF)
colloidal gold 12 nm AffiniPure^®^donkey anti-goat IgG (H + L)	Jackson ImmunoResearch	705-205-147	AB_2340418	1:50 (IEM)
colloidal gold 6 nm donkey anti-goat IgG (H + L)	Aurion	806.333	NR	1:40 (IEM)
Alexa Fluor®594 AffiniPure® donkey antichicken IgY (IgG) (H+L)	Jackson ImmunoResearch	703-585-155	AB_2340377	1:500 (IF)

### Image processing and data analysis

Acquired microphotographs were processed using ImageJ (U.S. NIH, Bethesda, MD, USA; RRID: SCR_003070), Adobe Illustrator (version 29.0) (RRID: SCR_010279), and Adobe Photoshop (version 26.4) (RRID: SCR_014199) (Adobe Systems, San Jose, CA, USA). Modifications to images were limited to cropping and adjustment of brightness and contrast.

### LCM and RNA extraction

Rapidly frozen, isolated brain tissues were cut using cryostat at −23 °C and processed according to the protocol described by Garcia-Ovejero et al. (2018). In brief, 16 μm-thick coronal sections of the brain were transferred to PEN-Membrane slides (Leica Microsystems, #11505189). After drying for 10 min at 40 °C, the samples were fixed in 70% EtOH for 1 min, washed in DEPC-treated water, stained with 0.1% Toluidine Blue dye for 2 min, and washed again. Samples were transferred to 70% EtOH for 1 min and then to 100% EtOH for 30 s. Prepared tissue samples were dried for 15 min at 40 °C and processed using a Leica LMD6500 microscope equipped with LMD Software Laser Microdissection (Leica Microsystems). Four regions were collected for further gene expression analyses: (1) the SCO, (2) the hippocampus (vascularized region), (3) the choroid plexus of the third ventricle (CHP) (vascularized region/potential source of PECAM-1), and (4) the ependymal cells lining the third ventricle (avascular region). Samples were collected into the caps of 0.2 ml polymerase chain reaction (PCR) microtubes containing 20 µl of extraction solution from the Arcturus™ PicoPure™ RNA Isolation Kit (Applied Biosystems, Thermo Scientific, Vilnius, Lithuania, #KIT0204). The collected samples were subsequently incubated in the lids of the microtubes in a thermoblock for 30 min at 40 °C, centrifuged, and stored at −80 °C until the RNA isolation step. Total RNA for the expression analysis was isolated using the Arcturus™ PicoPure™ RNA Isolation Kit, according to the manufacturerʼs instructions. The isolated total RNA was eluted in 15 µl of elution buffer.

### Analysis of gene expression

Gene expression analysis was performed to evaluate the relative abundance of mRNA of the studied genes in the isolated brain regions. To analyze the relative expression, RT-qPCR with SYBR Green I detection was performed. PCR primers of the studied genes (Table 3) were designed according to their respective reference sequences by Primer-Blast (NIH, RRID: SCR_003095). For the *Pecam-1* gene, the primers were designed to target the region encoding extracellular domain of the protein, amplifying all known alternatively spliced mRNA variants. The cDNA for the RT-qPCR was prepared by reverse transcription of the extracted total RNA and a mixture of random hexamer primers and oligoT primers (random primer mix, New England Biolabs, Ipswich, MA, USA, #S1330S) using RevertAid Reverse Transcriptase (Thermo Scientific, Vilnius, Lithuania, #EP0442) following the manufacturerʼs instructions. The amplification reaction for each gene was performed in a 10 µl volume containing 1 x SG Master Mix (Xceed qPCR SG 2x Mix Lo-ROX, IAB, Prague, Czech Republic, #LPCR10501XL), 0.5 µM forward/reverse primer, and 3 µl of the sample containing the cDNA template. The amplification profile was as follows: 95 °C/2 min, 40 × (95 °C/10 s, 60–62 °C/25 s). After the last cycle, the melt curve analysis was performed. Single amplification product was confirmed for each gene/amplicon. The relative gene expression (relative quantity, RQ) of each analyzed gene/mRNA was calculated according to a standard curve, reflecting the PCR efficiency and dynamic range of the detection (Table 3). Samples for standard curve amplification were prepared by serial dilution of cDNA obtained from rat spinal cord or SCO. The Ct (Threshold cycle) values of all analyzed samples were within dynamic range of the analysis. The resulting RQ of the studied target genes (TG: *Pecam1*,* Sspo*,* Vim*) was normalized to the RQ of two reference genes (RG: *Eef1a1*,* Gapdh*), represented by a normalization factor (NF-RG) calculated as geometric mean of the respective RQs. The final normalized relative TG expression is expressed as the ratio of the relative TG expression to NF (RQ-TG/NF-RG).

**Table 3:** List of oligonucleotide primers used in analysis

Gene (GeneID, GenBank)	Gene (GenBank Acc. No)	Sequence: forward reverse	Product length Ta/efficiency
SCO-spondin (474348)	*Sspo* (NM_001007016)	5´-CTGAACCTCCTTTGCCCTGA-3´ 5´-CTTTGAGAACTCCCCACCCTG-3´	99 bp 60 °C/98.1%
Vimentin (81818)	*Vim* (NM_031140)	5´-TGATGTCCGCCAGCAGTATG-3´ 5´-TCAGAGAGGTCAGCAAACTTGG-3´	90 bp 62 °C/90.5%
Platelet endothelial cell adhesion molecule 1 (29583)	*Pecam-1* (NM_001439600)	5´-AGGAAGAGACGGTGTTGTCAC-3´ 5´-GGTTTGGAGAGCATTTCGCAC-3´	151 bp 62 °C/81.3%
Eukaryotic translation elongation factor 1 alpha 1 (171361)	*Eef1a1* (NM_175838)	5´-TGCTGGAGCCAAGTGCTAAT-3´ 5´-GTGCCAATGCCGCCAATTTT-3´	181 bp 60 °C/83%
Glyceraldehyde-3-phosphate dehydrogenase (24383)	*Gapdh* (NM_017008)	5´-AGACAGCCGCATCTTCTTGT-3´ 5´-TGATGGCAACAATGTCCACT-3´ (Bangaru et al. 2012)	142 bp 60 °C/96.6%

### Statistics

For statistical analysis and graph generation, we used GraphPad Prism software (version 10.3.1, GraphPad Software, San Diego, CA, USA, RRID: SCR_002798). The data were analyzed using one-way analysis of variance (ANOVA) followed by the Tukey–Kramer post hoc test for multiple comparisons. A difference between groups was considered statistically significant if the p value was less than 0.05.

## Results

### Reissner´s fiber of rat CNS is immunoreactive to anti-PECAM-1 antibody

**Fig. 1:**
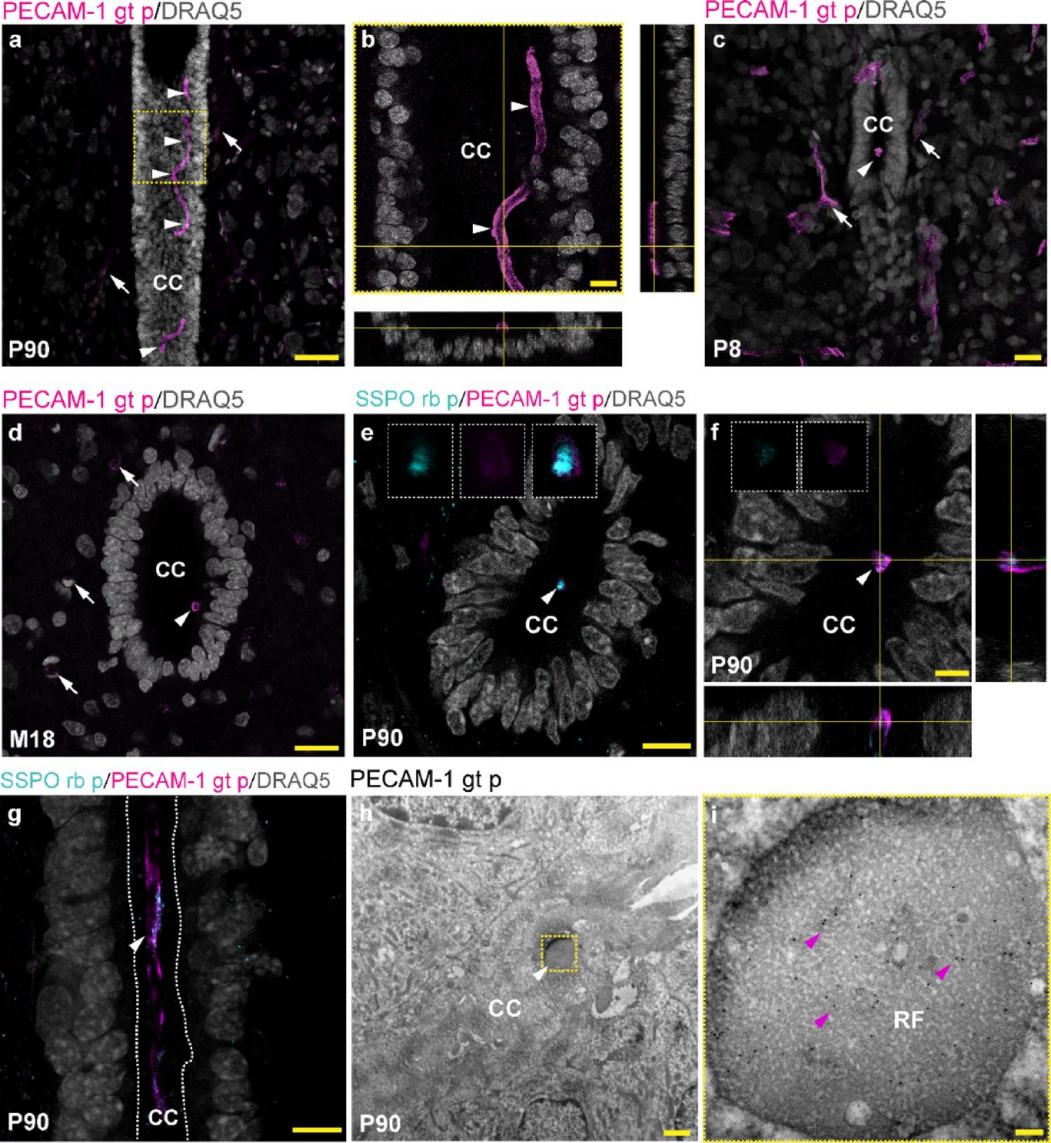
Immunoreactivity of blood vessels and RF to goat anti-PECAM-1 antibody in rat spinal cord. PECAM-1^+^ (magenta, goat polyclonal anti-PECAM-1 antibody) parenchymal blood vessels (white arrows) and the elongated structure corresponding to RF (white arrowheads) present in the CC of spinal cord (**a**, Z-stack); the yellow dashed line square in (**a**) corresponds to the CC lining region; PECAM-1^+^ RF (magenta, white arrowheads) is viewed in the single optical section and in orthogonal projections of the Z-stack (yellow crossed lines) (**b**); PECAM-1^+^ parenchymal blood vessels (magenta, white arrows) and RF (white arrowheads) in the coronal section of spinal cord at P8 (**c**) and M18 (**d**); co-localization of SCO-spondin (cyan, rabbit polyclonal anti-SSPO antibody) and PECAM-1 (magenta) in RF (white arrowhead) magnified in white dashed line rectangles in coronal section of spinal cord (**e**); detail of SSPO^+^ (cyan) and PECAM-1^+^ (magenta) RF (white arrowhead) in the single optical section and in orthogonal projections of the Z-stack (yellow crossed lines), magnified in white dashed line squares (**f**); co-localization of SCO-spondin (cyan) and PECAM-1 (magenta) in RF (white arrowhead) in the longitudinal section of spinal cord, white dashed lines border the CC lumen (**g**, Z-stack); nuclei of cells (gray, DRAQ5) in (**a–g**); ultrathin section of spinal cord shows ependymal lining and the CC lumen with RF (white arrowhead) depicted in the yellow dashed line square (**h**) magnified in (**i**) with gold nanoparticles (magenta arrowheads) corresponding to goat anti-PECAM-1 antibody binding to RF; representative images of lumbar spinal cord of rats at postnatal day 8 (P8); 90 days (P90), and 18 months (M18); scale bar: **a** = 50 μm;** b, e, g** = 10 μm; **c, d** = 20 μm; **f** = 5 μm, **h** = 2 μm; **i** = 100 nm

Only a limited number of antibodies are available to visualize RF in the rodent CNS using immunolabeling, such as anti-SCO-spondin or bovine AFRU antisera (Corales et al. 2022; Muñoz et al. 2019; Rodríguez et al. 1984; Sterba et al. 1981). Our current analyses indicate that anti-PECAM-1 antibodies may serve as an alternative to visualizing the RF in the rat CNS. We observed that the elongated structure present in the lumen of the CC in the spinal cord of P90 rats is immunoreactive to polyclonal goat anti-PECAM-1 antibody (RRID: AB_2161028, gt p) (Fig. 1a, b). The Z-stacks and orthogonal projections of the ependymal lining region proved that an elongated PECAM-1-positive structure is located in the CC lumen over the apical surface of ependymal cells. Since the ependymal lining surrounding the CC is an avascular region, the extracellular structure of approximately 4 μm in diameter should correspond to RF (Fig. 1a, b). Indeed, we observed that the anti-PECAM-1 antibody binds to RF in the neonatal stage (P8) (Fig. 1c), as well as other studied time points, including senescence (M18) (Fig. 1d). Although we showed PECAM-1^+^ RF of the lumbar spinal cord, it was also identified using anti-PECAM-1 antibody in other regions, from cervical to sacral segments (Supplementary Fig. 1). To prove that identified PECAM-1^+^ structure present in CC lumen corresponds to RF, co-localization of anti-PECAM-1 and anti-SSPO antibodies detecting SCO-spondin was applied. According to our results, PECAM-1^+^ structure corresponds to RF (Fig. 1e). While SSPO labelling is apparent mostly in the inner part of RF, PECAM-1 was detected mostly on its surface (Fig. 1f, g). Immunogold labeling revealed that globular electron-dense material present in the lumen of the CC in coronal sections of the spinal cord corresponds to RF (Fig. 1h). According to immunogold labeling superficial as well as the inner regions of RF are immunoreactive to the anti-PECAM-1 antibody (Fig. 1i).

### Only the apical surface of SCO cells is immunoreactive to anti-PECAM-1 antibody

**Fig. 2:**
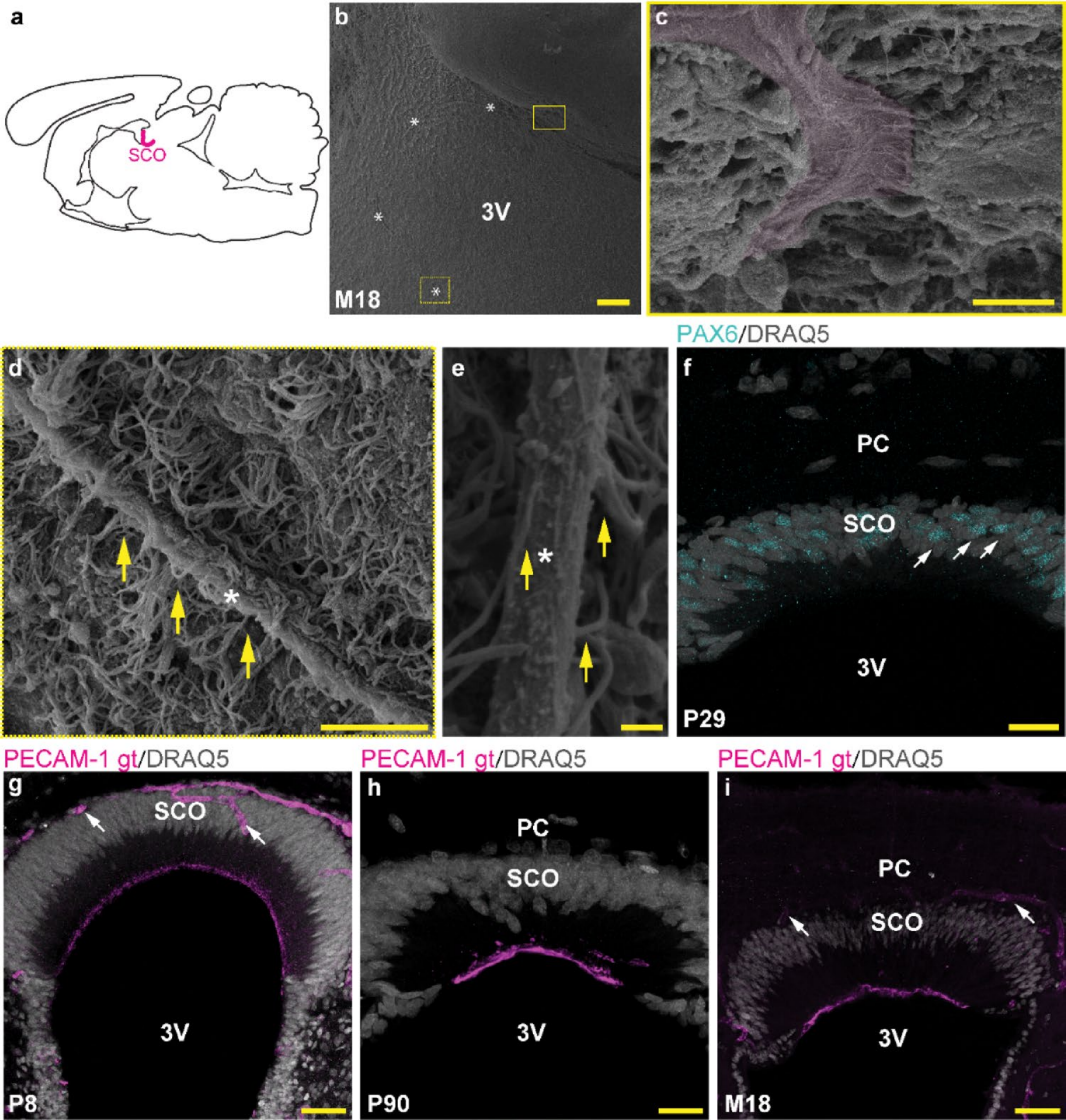
The apical surface of SCO cells and nearby vasculature is immunoreactive to anti-PECAM-1 antibody. Schematic representation of a sagittal section of the brain with the SCO in the third ventricle (3 V) (**a**); a panoramic view showing ependymal lining in the 3 V with a thread-like structure corresponding to RF (*) lying on the surface of the ependymal cells (**b**); the yellow line rectangle in (**b**) represents the region with extracellular material on the surface of the SCO which corresponds to the initial part of RF (pre-RF, magenta pseudo-color) (**c**); the yellow dashed line rectangle in (**b**) represents detail of RF (*) in 3 V, where RF is covered by cilia of multiciliated ependymal cells (yellow arrows) (**d**); RF (*) in the CC of the spinal cord in contact with several cilia of ependymal cells (yellow arrows) (**e**); PAX6^+^ nuclei (cyan color, rabbit monoclonal anti-PAX6 antibody) of SCO cells, PC corresponds to posterior commissure (**f**); goat anti-PECAM-1 antibodies (magenta) bind to the apical surface of SCO cells and nearby vasculature (white arrows) in neonatal (P8) (**g**), young adult (P90) (**h**), and senescent (M18) rats (**i**); nuclei of cells (gray, DRAQ5) in (**f–i**). Representative images from various age groups (P8, P29, P90, and M18); scale bar: **b** = 100 μm; **c, d** = 10 μm; **e** = 1 μm; **f** = 20 μm; **g** = 50 μm, **h** = 20 μm, **i** = 50 μm

As previously mentioned, RF is formed by the aggregation of extracellular material produced by SCO cells. Thus, we focused on the identifying the SCO and RF in the brain ventricular system using SEM, and analyzed the potential immunoreactivity of SCO cells to an anti-PECAM-1 antibody. SCO cells were localized in the dorso-caudal region of the third ventricle (3 V) in association with the long extracellular structure corresponding to RF (Fig. 2a, b). A mass of extracellular material, which could correspond to pre-RF was identified on the surface of the ciliated cells in the initial region of RF (Fig. 2c). The RF is covered by cilia from multiciliated ependymal cells of the 3 V (Fig. 2d) and extends caudally to the CC of the spinal cord (Fig. 2e). In coronal sections, the SCO region appears like a groove formed by numerous elongated cells with a wide central region, narrowing rostrally and caudally. Similar to what was previously observed in the mouse CNS, the SCO of postnatal rats harbors a subpopulation of PAX6^+^ cells (Fig. 2f). As expected, blood vessels surrounding the SCO cells are immunoreactive to the anti-PECAM-1 antibody. In addition, unambiguous PECAM-1 immunolabeling was also identified in the apical region of the SCO cells facing the lumen of the third ventricle. Prominent labeling of PECAM-1 in this region was observed at all studied time points (Fig. 2g–i).

**Fig. 3:**
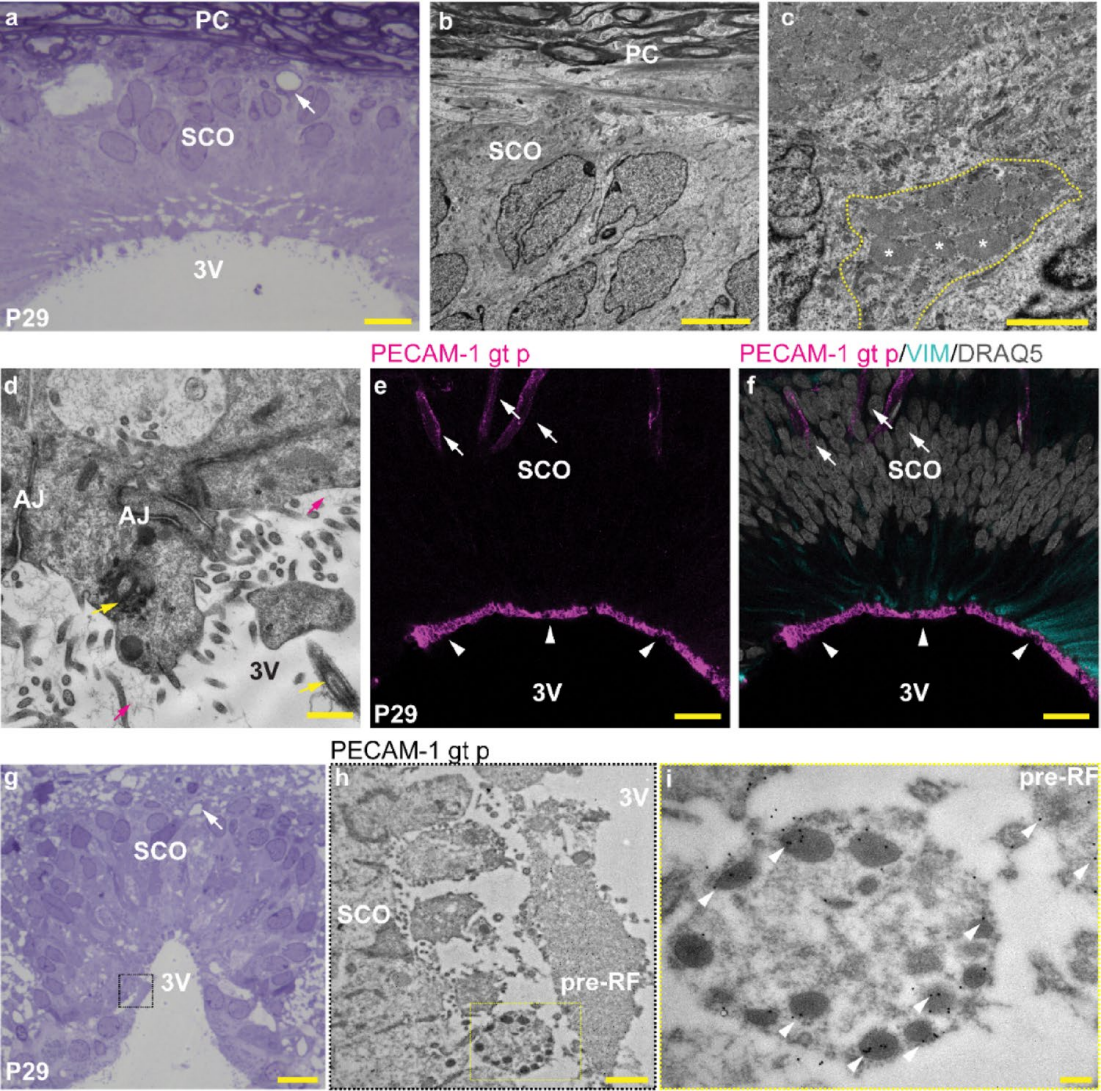
Blood vessels and the apical surface of SCO cells are immunoreactive to anti-PECAM-1. Toluidine Blue stained section showing SCO cells, PC, and 3 V (**a**); invaginations of the nuclear envelope and euchromatin are visible in nuclei of SCO cells close to the PC (**b**); wide cisterns of RER (white asterisks) are present in the central region of SCO cell indicated by yellow dashed line (**c**); apical protrusions of SCO cells with microvilli (yellow arrowheads) and cilia (yellow arrows), small vesicle in the apical cytoplasm and fine filamentous material in 3 V (magenta arrows), adherent junctions (AJ) (**d**); PECAM-1 (magenta, anti-PECAM-1 antibody) is present in the blood vessels (white arrows) and in the apical region of SCO cells (white arrowheads) (**e**); PECAM-1 (magenta, anti-PECAM-1 antibody) is present in the very apical region of SCO cells (white arrowheads) above the intermediate filaments (cyan, anti-vimentin antibody, VIM) (**f**); panoramic view of SCO cells in Toluidine Blue-stained coronal sections of caudal regions of the SCO (**g**); region depicted by black dashed line square in (**g**) reveals region of apical protrusions and pre-RF magnified in (**h**); yellow dashed line rectangle in (**h**) magnified in (**i**) depicts gold nanoparticles (white arrowheads) corresponding to anti-PECAM-1 antibody in the electron-dense vesicles of the apical protrusion of SCO cells and in pre-RF (white arrowheads) located in the lumen of the 3 V. Representative images of P29 rat brain; scale bar: **a** = 10 μm; **b** = 5 μm; **c, d** = 1 μm; **e** = 50 μm; **f** = 50 μm; **g** = 10 μm; **h** = 1 μm; **i** = 200 nm

Ultrastructural analysis of the SCO and RF in the brain and spinal cord of P29 rats revealed that densely organized, elongated secretory cells of the SCO have pale, basally located nuclei, which mainly contain euchromatin. The nuclei are round to elongated with a deeply invaginated nuclear envelope (Fig. 3a, b). The cytoplasm of the SCO cells contains numerous wide cisterns of rough endoplasmic reticulum (RER) filled with electron-dense material (Fig. 3c). Small vesicles are identified in the apical cytoplasm of the SCO cells. Fine filamentous material is present in the lumen of 3 V, close to the microvilli and cilia of the luminal surface of the SCO cells (Fig. 3d). Similar to other time points studied, PECAM-1 was identified in the blood vessels located in the basal regions of the SCO near the posterior commissure (PC). PECAM-1, present in the apical regions of SCO cells, corresponds by its position to the apical protrusions directed to the lumen of the 3 V. This is the most apical region of SCO cells over the intermediate filaments and first adherent junctions (Fig. 3d–f). Immunogold labeling supported the presence of PECAM-1 in the apical protrusions of the SCO cells, particularly localized in the electron-dense vesicles. Interestingly, lower regions of the SCO cell bodies were PECAM-1 negative (data not shown). PECAM-1 was also identified in the amorphous extracellular material present in the lumen of the 3 V, which corresponds to pre-RF (Fig. 3g–i).

### A different pattern of immunolabeling using various anti-PECAM-1 antibodies is observed in RF and the SCO of rats

**Fig. 4:**
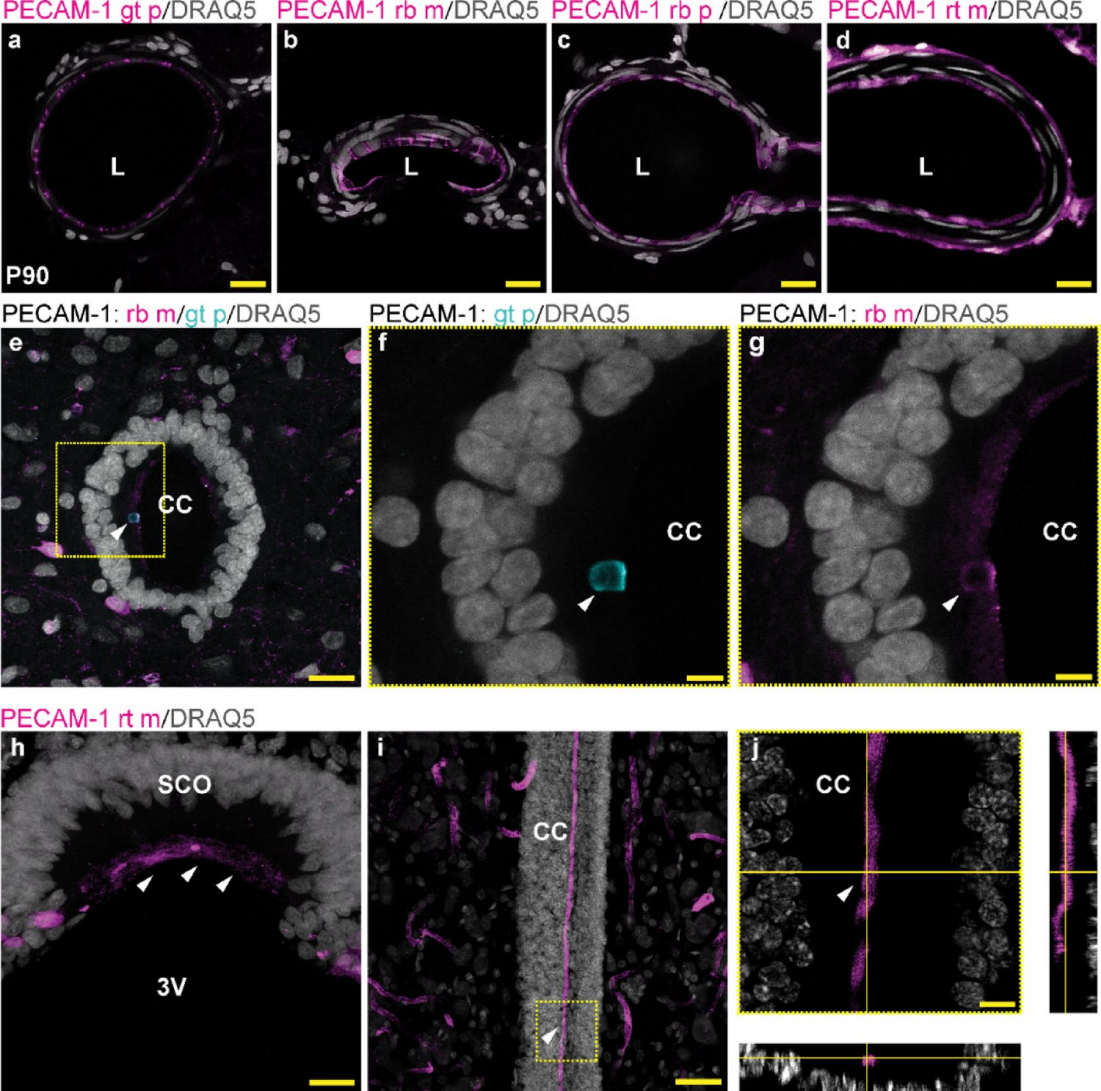
Pattern of PECAM-1 immunolabeling using antibodies recognizing different regions of PECAM-1. Pattern of PECAM-1 in the *arteria spinalis* using goat polyclonal anti-PECAM-1 (**a**), rabbit monoclonal anti-PECAM-1 (**b**), rabbit polyclonal anti-PECAM-1 (**c**), and rat monoclonal anti-PECAM-1 antibodies (**d**); goat polyclonal anti-PECAM-1 antibody (cyan) and rabbit monoclonal anti-PECAM-1 (magenta) detect RF (white arrowhead) (**e**); the yellow dashed line square in (**e**) corresponds to the region magnified in (**f–g**); apical surface of SCO cells (white arrowheads) immunolabeled by rat monoclonal anti-PECAM-1 (magenta) (**h**) and RF (white arrowhead) in the CC of spinal cord in the Z-stack (**i**); the region indicated by yellow dashed line square magnified in (**j**) in single optical section and orthogonal views contains RF present in the CC lumen surrounded by ependymal cells (yellow crossed lines in the Z-projection) (**j**). Representative images of lumbar spinal cord and brain of P90 rats; L in (**a–d**) corresponds to the lumen of *arteria spinalis*; scale bar: **a–e** = 20 μm; **f–g** = 5 μm; **h** = 20 μm; **i** = 50 μm; **j** = 10 μm

To confirm the application of PECAM-1 antibodies in immunolabeling of RF, we tested several other commercially available anti-PECAM-1 antibodies, including rabbit monoclonal (RRID: AB_2905525; rb m), rabbit polyclonal (RRID: AB1_10002513; rb p), and rat monoclonal antibody (RRID: AB_467202; rt m). As already shown, immunolabeling using goat anti-PECAM-1 antibody (RRID: AB_2687506; gt p) clearly indicates the presence of PECAM-1 in blood vessels, in RF, and in apical protrusions of SCO cells. All other tested antibodies bind to PECAM-1 of endothelial cells (Fig. 4a–d); however, their affinity to RF and SCO cells varies. In comparison to the presented immunolabeling using gt p anti-PECAM-1 antibody, only a faint signal on RF was acquired using rb m anti-PECAM-1 antibody if an antigen retrieval step was added to the immunolabeling protocol (10 min in citrate buffer, pH 6.0 at 95–98 °C before immunolabeling) (Fig. 4e–g). Interestingly, PECAM-1 was not detected in RF or SCO cells using the rb p anti-PECAM-1 antibody. On the other hand, clear PECAM-1 immunolabeling was observed in RF and SCO cells using rt m anti-PECAM-1 antibody corresponding to the pattern of gt p anti-PECAM-1 (Fig. 4h–j). Thus, our analyses reveal that the rb p anti-PECAM-1 antibody is not suitable for visualizing RF or SCO cells. Although all tested anti-PECAM-1 antibodies recognize the same glycoprotein, they bind to different sites on PECAM-1. The rb p anti-PECAM-1 antibody, which did not bind to RF or SCO cells, recognizes the cytoplasmic region of PECAM-1, while gt p and rb m antibodies bind to the extracellular region of PECAM-1. In case of rt m anti-PECAM-1 antibody, which shows positivity against RF and SCO cells, the manufacturer does not specify the target site.

### *PECAM-1* mRNA is not detected in SCO cells

**Fig. 5:**
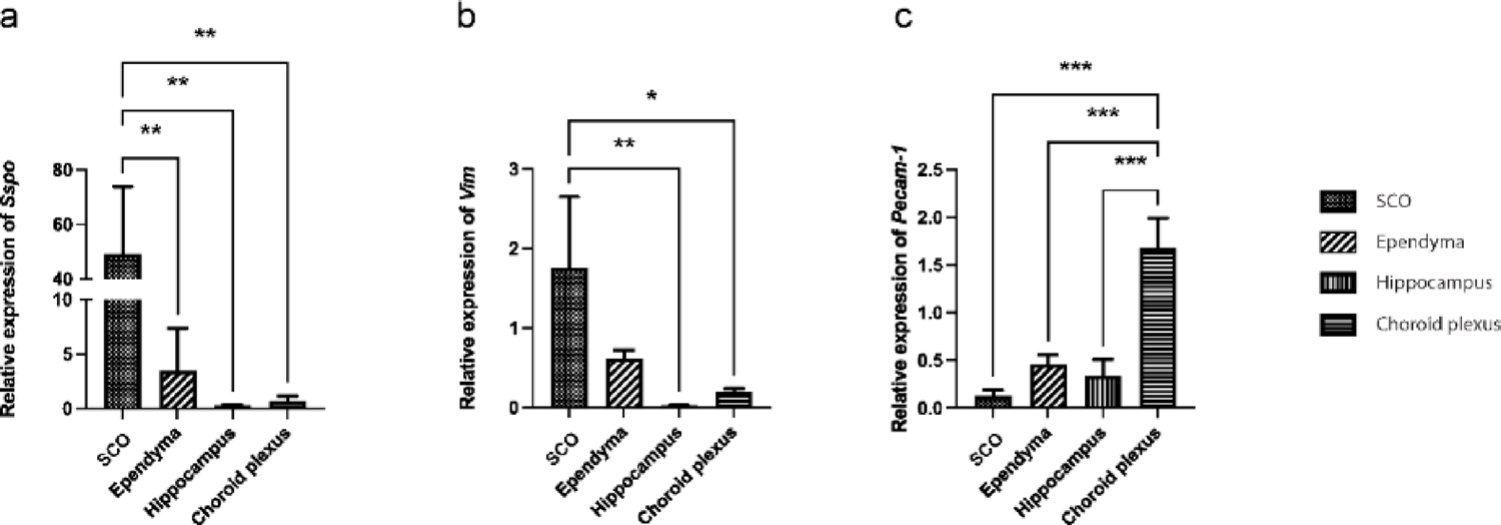
Relative expression of *Sspo*, *Vimentin (Vim)*, and *Pecam-1* genes in the studied regions of the rat brain. Significantly high expression of the *Sspo* gene in the SCO is shown in comparison to the other studied regions (**a**); high expression of *Vimentin* in the SCO and ependymal lining (**b**); and significantly high expression of the *Pecam-*1 gene in highly vascularized choroid plexus in comparison to other studied regions (**c**). Relative gene expression was analyzed by RT-qPCR and calculated according to a standard curve. The expression is normalized by Normalization factor based on expression of *Eef1a1* and *Gapdh* genes. The values represent an average ± SD obtained from 3 brain samples isolated from rats at the age of P90. Statistical significance: * *p* ≤ 0.05; ** *p* ≤ 0.01; *** *p* ≤ 0.001

Since immunogold labeling revealed PECAM-1 only in the apical protrusions but not in the other regions of SCO cells, we analyzed whether *Pecam-1* is expressed by the SCO cells in samples isolated by the LCM technique. Since PECAM-1 is typically present in blood vessels, we also isolated other brain regions as a control samples, such as the hippocampus and choroid plexus, both of which represent vascularized areas. In addition, we isolated the ependymal lining of the 3 V, which represents an avascular region. To verify the accuracy of SCO isolation, we also analyzed the expression of the *Sspo* gene encoding SCO-spondin, highly produced by SCO cells, and the expression of *Vimentin* encoding the intermediate filament Vimentin in the cytoskeleton of SCO cells. The quantitative analysis enabled us to identify regions expressing significant levels or only traces of the analyzed transcripts. As expected, our analysis showed that *Sspo* is highly expressed in SCO cells. Conversely, only traces of *Sspo* mRNA were detected in the remaining dissected regions. This could be due to minor contamination of the samples with cells of the SCO, especially the neighboring ependymal lining (Fig. 5a). *Vimentin* mRNA was detected in both SCO cells and ependymal cells, while lower expression was detected in the vascularized regions (Fig. 5b). On the other hand, *Pecam-1* mRNA was detected predominantly in the choroid plexus with a significant difference compared to SCO samples (Fig. 5c), indicating that SCO cells are not the primary source of PECAM-1 targeted by the anti-PECAM-1 antibody used for immunolabeling. Considering that several variants of *Pecam-1* mRNA arise from alternative splicing, the oligonucleotide primers designed for detecting *Pecam-1* mRNA were targeted to the sequence encoding the extracellular region present in all known *Pecam-1* variants.

## Discussion

Although the SCO and RF have been intensively studied during the past decades, their functions remain only partly understood. RF is considered an adhesive structure, which is formed by the aggregation of proteinaceous material secreted by SCO cells. Anatomical localization and ultrastructural analyses of RF and SCO cells presented in this study reveal the typical morphology of the SCO–RF in the CNS of rats (Corales et al. 2022; Guerra et al. 2015; Muñoz et al. 2019; Vio et al. 2008). We observed a filamentous mass of material covering the apical surface of SCO cells in the 3 V, corresponding to the pre-RF region. The structure gradually changes its organization to become the thread-like RF, covered by numerous cilia of ependymal cells, which aligns with previous studies (Muñoz et al. 2019; Nualart et al. 2023; Woollam and Collins 1980). According to the work of Schoebitz et al. (1986), aggregating material produced by SCO cells may be identified in the brain of rats at P1 and in the spinal cord at P2. We identified RF in the spinal cord at P8, which aligns with the observations of the first appearance of a proper RF in the spinal cord at P6. We observed that RF of rats is immunoreactive to anti-SSPO antibody, and surprisingly also to anti-PECAM-1 antibodies binding to PECAM-1, a characteristic typical of endothelial cells, platelets, and certain types of blood cells (Newman et al. 1990; Simmons et al. 1990). Interestingly, the PECAM-1^+^ structure corresponding to RF was identified in the lumen of the CC of the spinal cord at all studied time points, including the neonatal period (P8) and in senescence (M18). PECAM-1 represents a glycoprotein consisting of three regions: a highly glycosylated extracellular N-terminal region composed of six immunoglobulin (Ig)-like domains, a single short transmembrane-spanning domain, and a C-terminal cytoplasmic region (Newman et al. 1990; Newton et al. 1999). The cytoplasmic region of PECAM-1 is highly variable due to extensive alternative splicing of the encoding *Pecam-1* mRNA, resulting in several truncated isoforms with altered functions (reviewed in Newman and Newman 2003). On the other hand, there is no record regarding the rearrangement of the extracellular region of the protein. Nevertheless, several reports describe the formation of soluble variants of PECAM-1 (sPECAM-1), which may arise by alternative splicing, specifically skipping exon 9 that encodes the transmembrane domain. This results in the formation of sPECAM-1, which possesses extracellular and cytoplasmic domains only (Goldberger et al. 1994). In addition, truncated soluble sPECAM-1 variants may arise by proteolytic cleavage of the extracellular region of PECAM-1 on endothelial or blood cells, particularly leukocytes and platelets, shedding the sPECAM-1 into the plasma or CSF during apoptosis or in pathological conditions (Fornasa et al. 2010; Ilan et al. 2001; Kalinowska and Losy 2006; Magnoni et al. 2025; Tran-Dinh et al. 2022). According to our results, the extracellular region of the PECAM-1 protein could be incorporated into the structure of RF, since only anti-PECAM-1 antibodies recognizing the extracellular region of PECAM-1 bind to RF and the apical surface of SCO cells (goat polyclonal, RRID: AB_2687506, and rabbit monoclonal, RRID: AB_2905525, antibodies). While the goat polyclonal antibody unambiguously labels RF and SCO cells, the rabbit monoclonal antibody binds to RF only weakly, potentially due to its higher specificity compared to the polyclonal antibody, which may cover a larger part of the extracellular region. In addition, we applied a rat monoclonal anti-PECAM-1 antibody (RRID: AB_467202), which also detects SCO and RF, similar to the goat polyclonal anti-PECAM-1 antibody. However, the manufacturer does not specify the PECAM-1 region detected by the antibody. The pattern of labeling of RF or SCO cells using antibodies against the extracellular region of PECAM-1 did not correspond to the labeling using rabbit polyclonal anti-PECAM-1 antibody that recognizes the cytoplasmic region of PECAM-1 (RRID: AB1_10002513). No signal of this antibody was detected in the RF or apical region of SCO cells.

Therefore, we assume that reliable immunolabeling of RF and SCO cells in the rat CNS using anti-PECAM-1 antibodies could depend on the domain targeted by the antibody and thus should be carefully selected. Although the SCO is considered the main source of components for RF, PECAM-1 was found only in the apical regions of the SCO cells, at the level above the intermediate filaments and first adherent junction, while the lower parts of the cells were negative. PECAM-1 is localized to electron-dense vesicles of apical protrusions of SCO cells and to the material of pre-RF, which suggests that the glycoprotein could be incorporated into RF during the early stages of its formation, rather than attached to its surface as is assumed in the case of the monoamines (Caprile et al. 2003). Similar results were obtained by Muñoz et al. (2019), who described the presence of glycoprotein Gal-1 on the surface of RF. Gal-1 is identified only in the most apical region of the SCO cells and in the RF structure. Although the exact mechanism behind the unique way glycoproteins are incorporated into RF is not clear, according to the authors, Gal-1 plays an important role; it binds sialylated glycans attached to SCO-spondin dimers, forming oligomers identified as 10 nm microfilaments, which are subsequently packed into fibrils via a mechanism facilitated by clusterin. The source of Gal-1 is a discrete subpopulation of ependymal cells lining the 3 V adjacent to the SCO. The target itself or the origin of PECAM-1 identified in RF in our study using anti-PECAM-1 antibodies remains an important question. Since a significant amount of *Pecam-1* mRNA was not detected in SCO cells, with PECAM-1 present only in the apical region, it is possible to infer that the SCO is not the primary source of PECAM-1 similar as Gal-1. In addition, the presence of PECAM-1 in the CSF of intact rats is uncertain, but elevated levels of sPECAM-1 have been detected in the CSF of humans after ischemic stroke (Zaremba and Losy 2002). We assume that vascular choroid plexus, which is a main producer of CSF, a site of entry of immune cells from the bloodstream, and a hub of immune activity (Xu et al. 2023), or potentially another region of the ventricular system, could be the source of PECAM-1. To understand its origin, further analysis is required. In addition, another question arises: If PECAM-1 is truly present in RF, what is its function in the SCO–RF? Kitazume et al. (2014) demonstrated that binding of PECAM-1 to sialic acid leads to transduction of anti-apoptotic signals to vascular endothelial cells. Potentially, the ability of PECAM-1 to bind sialic acid residues suggests that it could represent another key player in RF aggregation, similar to Gal-1. Our observations also highlight another, more general issue with immunolabeling, namely the lack of information on the immunogen or target of commercially available antibodies. This information is often underestimated, but in many cases, it prevents an objective assessment of the obtained data, sometimes in the context of new knowledge (e.g. identification of new unknown isoforms).

## Conclusion

Our study revealed that PECAM-1 is another marker of the SCO–RF in the CNS of rats. According to our results, the truncated extracellular region of PECAM-1 could be embedded in RF and the apical surface of SCO cells, which can be visualized using anti-PECAM-1 antibodies. Further analyses are required to verify the application of our findings in other animal species that form RF and to elucidate the origin and function of PECAM-1 in the SCO–RF of rats.

## Supplementary Information

Below is the link to the electronic supplementary material.

Supplementary Material 1

## Data Availability

No datasets were generated or analysed during the current study.

## References

[CR1] Bangaru ML, Park F, Hudmon A, McCallum JB, Hogan QH (2012) Quantification of gene expression after painful nerve injury: validation of optimal reference genes. J Mol Neurosci 46(3):497–504. 10.1007/s12031-011-9628-x 21863315 10.1007/s12031-011-9628-x PMC3273664

[CR2] Bellegarda C, Zavard G, Moisan L, Brochard-Wyart F, Joanny J-F, Gray RS, Cantaut-Belarif Y, Wyart C (2023) The Reissner fiber under tension in vivo shows dynamic interaction with ciliated cells contacting the cerebrospinal fluid. Elife 12:e86175. 10.7554/elife.86175 37772792 10.7554/eLife.86175 PMC10617989

[CR3] Cantaut-Belarif Y, Sternberg JR, Thouvenin O, Wyart C, Bardet P-L (2018) The Reissner fiber in the cerebrospinal fluid controls morphogenesis of the body axis. Curr Biol 28(15):2479-2486e.e4. 10.1016/j.cub.2018.05.079 30057305 10.1016/j.cub.2018.05.079 PMC6089837

[CR4] Cantaut-Belarif Y, Orts Del’Immagine A, Penru M, Pézeron G, Wyart C, Bardet P-L (2020) Adrenergic activation modulates the signal from the Reissner fiber to cerebrospinal fluid-contacting neurons during development. Elife 9:e59469. 10.7554/eLife.59469 33048048 10.7554/eLife.59469 PMC7591253

[CR5] Caprile T, Hein S, Rodríguez S, Montecinos H, Rodríguez E (2003) Reissner fiber binds and transports away monoamines present in the cerebrospinal fluid. Mol Brain Res 110(2):177–192. 10.1016/S0169-328X(02)00565-X 12591155 10.1016/s0169-328x(02)00565-x

[CR6] Castenholz A, Zöltzer H (1980) Formation and morphology of reissner’s fibre in primates: a scanning electron microscopic study. Cell Tissue Res 207(1):43–53. 10.1007/BF00239328 6771014 10.1007/BF00239328

[CR7] Chen I-L, Lu K-S, Lin H-S (1973) Electron microscopic and cytochemical studies of the mouse subcommissural organ. Z Für Zellforschung Und Mikroskopische Anatomie 139(2):217–236. 10.1007/BF00306523 10.1007/BF00306523 4123204

[CR8] Corales LG, Inada H, Hiraoka K, Araki S, Yamanaka S, Kikkawa T, Osumi N (2022) The subcommissural organ maintains features of neuroepithelial cells in the adult mouse. J Anat 241(3):820–830. 10.1111/joa.13709 35638289 10.1111/joa.13709 PMC9358730

[CR9] Didier R, Meiniel R, Meiniel A (1992) Monoclonal antibodies as probes for the analysis of the secretory ependymal differentiation in the subcommissural organ of the chick embryo. Dev Neurosci 14(1):44–52. 10.1159/000111646 1600879 10.1159/000111646

[CR10] Diederen JHB (1970) The subcommissural organ of *Rana temporaria* L. Z Für Zellforschung Und Mikroskopische Anatomie 111(3):379–403. 10.1007/BF00342489 10.1007/BF00342489 4099828

[CR11] Duvernoy HM, Risold P-Y (2007) The circumventricular organs: an atlas of comparative anatomy and vascularization. Brain Res Rev 56(1):119–147. 10.1016/j.brainresrev.2007.06.002 17659349 10.1016/j.brainresrev.2007.06.002

[CR12] Feng D, Nagy JA, Pyne K, Dvorak HF, Dvorak AM (2004) Ultrastructural localization of platelet endothelial cell adhesion molecule (PECAM-1, CD31) in vascular endothelium. J Histochem Cytochem 52(1):87–101. 10.1177/002215540405200109 14688220 10.1177/002215540405200109

[CR13] Fernández-Llebrez P, Pérez J, Cifuentes M, Alvial G, Rodríguez EM (1987) Immunocytochemical and ultrastructural evidence for a neurophysinergic innervation of the subcommissural organ of the snake natrix Maura. Cell Tissue Res 248(2):473–478. 10.1007/BF00218215 2884036 10.1007/BF00218215

[CR14] Fornasa G, Groyer E, Clement M, Dimitrov J, Compain C, Gaston A-T, Varthaman A, Khallou-Laschet J, Newman DK, Graff-Dubois S, Nicoletti A, Caligiuri G (2010) TCR stimulation drives cleavage and shedding of the ITIM receptor CD31. J Immunol 184(10):5485–5492. 10.4049/jimmunol.0902219 20400708 10.4049/jimmunol.0902219 PMC3110943

[CR15] Galarza M (2002) Evidence of the subcommissural organ in humans and its association with hydrocephalus. Neurosurg Rev 25(4):205–215. 10.1007/s10143-002-0208-y 12172724 10.1007/s10143-002-0208-y

[CR16] Garcia-Ovejero D, Paniagua-Torija B, Arevalo-Martin A, Navarro-Galve B, Molina-Holgado E (2018) Laser-capture microdissection for the analysis of rat and human spinal cord ependyma by qPCR. In: Murray G (ed) Laser capture microdissection methods in molecular biology, vol 1723. Humana Press, New York, NY, pp 285–318. 10.1007/978-1-4939-7558-7_17 10.1007/978-1-4939-7558-7_17 29344868

[CR17] Gobron S, Monnerie H, Meiniel R, Creveaux I, Lehmann W, Lamalle D, Dastugue B, Meiniel A (1996) SCO-spondin: a new member of the thrombospondin family secreted by the subcommissural organ is a candidate in the modulation of neuronal aggregation. J Cell Sci 109(5):1053–1061. 10.1242/jcs.109.5.1053 8743952 10.1242/jcs.109.5.1053

[CR18] Gobron S, Creveaux I, Meiniel R, Didier R, Dastugue B, Meiniel A (1999) SCO-spondin is evolutionarily conserved in the central nervous system of the chordate phylum. Neuroscience 88(2):655–664. 10.1016/S0306-4522(98)00252-8 10197783 10.1016/s0306-4522(98)00252-8

[CR19] Gobron S, Creveaux I, Meiniel R, Didier R, Herbet A, Bamdad M, El Bitar F, Dastugue B, Meiniel A (2000) Subcommissural organ/Reissner’s fiber complex: characterization of SCO-spondin, a glycoprotein with potent activity on neurite outgrowth. Glia 32(2):177–191. 11008217 10.1002/1098-1136(200011)32:2<177::aid-glia70>3.0.co;2-v

[CR20] Goldberger A, Middleton KA, Oliver JA, Paddock C, Yan HC, DeLisser HM, Albelda SM, Newman PJ (1994) Biosynthesis and processing of the cell adhesion molecule PECAM-1 includes production of a soluble form. J Biol Chem 269(25):17183–17191. 10.1016/S0021-9258(17)32538-3 8006026

[CR21] Gonçalves-Mendes N, Simon-Chazottes D, Creveaux I, Meiniel A, Guénet J-L, Meiniel R (2003) Mouse SCO-spondin, a gene of the thrombospondin type 1 repeat (TSR) superfamily expressed in the brain. Gene 312:263–270. 10.1016/s0378-1119(03)00622-x 12909363 10.1016/s0378-1119(03)00622-x

[CR22] Grondona JM, Hoyo-Becerra C, Visser R, Fernández-Llebrez P, López-Ávalos MD (2012) The subcommissural organ and the development of the posterior commissure. Int Rev Cell Mol Biol 296:63–137. 10.1016/B978-0-12-394307-1.00002-3 22559938 10.1016/B978-0-12-394307-1.00002-3

[CR23] Guerra MM, González C, Caprile T, Jara M, Vío K, Muñoz RI, Rodríguez S, Rodríguez EM (2015) Understanding how the subcommissural organ and other periventricular secretory structures contribute via the cerebrospinal fluid to neurogenesis. Front Cell Neurosci 9(480):1–17. 10.3389/fncel.2015.00480 26778959 10.3389/fncel.2015.00480 PMC4689152

[CR24] Hayat MA (1972) Basic electron microscopy techniques. Van Nostrand Reinhold Company, New York. 119. ISBN 9780442784423

[CR25] Hoyo-Becerra C, López-Ávalos MD, Cifuentes M, Visser R, Fernández-Llebrez P, Grondona JM (2010) The subcommissural organ and the development of the posterior commissure in chick embryos. Cell Tissue Res 339(2):383–395. 10.1007/s00441-009-0899-2 20012322 10.1007/s00441-009-0899-2

[CR26] Ilan N, Mohsenin A, Cheung L, Madri JA (2001) PECAM-1 shedding during apoptosis generates a membrane‐anchored truncated molecule with unique signaling characteristics. FASEB J 15(2):362–372. 10.1096/fj.00-0372com 11156952 10.1096/fj.00-0372com

[CR27] Kalinowska A, Losy J (2006) PECAM-1, a key player in neuroinflammation. Eur J Neurol 13(12):1284–1290. 10.1111/j.1468-1331.2006.01640.x 17116209 10.1111/j.1468-1331.2006.01640.x

[CR28] Kitazume S, Imamaki R, Kurimoto A, Ogawa K, Kato M, Yamaguchi Y, Tanaka K, Ishida H, Ando H, Kiso M, Hashii N, Kawasaki N, Taniguchi N (2014) Interaction of platelet endothelial cell adhesion molecule (PECAM) with α2,6-Sialylated glycan regulates its cell surface residency and anti-apoptotic role. J Biol Chem 289(40):27604–27613. 10.1074/jbc.M114.563585 25135639 10.1074/jbc.M114.563585 PMC4183799

[CR29] Lu H, Shagirova A, Goggi JL, Yeo HL, Roy S (2020) Reissner fibre-induced urotensin signalling from cerebrospinal fluid-contacting neurons prevents scoliosis of the vertebrate spine. Biol Open 9(5):bio052027. 10.1242/bio.052027 32409296 10.1242/bio.052027 PMC7240301

[CR30] Magnoni M, Andreini D, Andreotti F, Latini R, Maseri A, Nicoletti A, Maggioni AP, Caligiuri G (2025) Leukocyte-shed soluble CD31 unmasks coronary disease in low-risk outliers and provides source-specific inflammatory signatures of vulnerable plaques. Atherosclerosis 407:120410. 10.1016/j.atherosclerosis.2025.120410 40543300 10.1016/j.atherosclerosis.2025.120410

[CR31] Meiniel A (2001) SCO-spondin, a glycoprotein of the subcommissural organ/reissner’s fiber complex: evidence of a potent activity on neuronal development in primary cell cultures. Microsc Res Tech 52(5):484–495. 11241859 10.1002/1097-0029(20010301)52:5<484::AID-JEMT1034>3.0.CO;2-0

[CR32] Meiniel O, Meiniel A (2007) The complex multidomain organization of SCO-spondin protein is highly conserved in mammals. Brain Res Rev 53(2):321–327. 10.1016/j.brainresrev.2006.09.007 17126404 10.1016/j.brainresrev.2006.09.007

[CR33] Meiniel R, Creveaux I, Dastugue B, Meiniel A (1995) Specific transcripts analysed by in situ hybridization in the subcommissural organ of bovine embryos. Cell Tissue Res 279(1):101–107. 10.1007/BF00300696 7895252 10.1007/BF00300696

[CR34] Meiniel O, Meiniel R, Lalloué F, Didier R, Jauberteau M-O, Meiniel A, Petit D (2008) The lengthening of a giant protein: when, how, and why? J Mol Evol 66(1):1–10. 10.1007/s00239-007-9055-3 18046595 10.1007/s00239-007-9055-3

[CR35] Meiniel, Molat J-L, Duchier-Liris N, Meiniel A (1990) Ontogenesis of the secretory epithelium of the bovine subcommissural organ. A histofluorescence study using lectins and monoclonal antibodies. Dev Brain Res 55(2):171–180. 10.1016/0165-3806(90)90198-8 2253320 10.1016/0165-3806(90)90198-8

[CR36] Molina B, Rodríguez EM, Peruzzo B, Caprile T, Nualart F (2001) Spatial distribution of Reissner’s fiber glycoproteins in the filum terminale of the rat and rabbit. Microsc Res Tech 52(5):552–563. 11241865 10.1002/1097-0029(20010301)52:5<552::AID-JEMT1040>3.0.CO;2-H

[CR37] Muñoz RI, Kähne T, Herrera H, Rodríguez S, Guerra MM, Vío K, Hennig R, Rapp E, Rodríguez E (2019) The subcommissural organ and the Reissner fiber: old friends revisited. Cell Tissue Res 375(2):507–529. 10.1007/s00441-018-2917-8 30259139 10.1007/s00441-018-2917-8

[CR38] Newman PJ, Newman DK (2003) Signal transduction pathways mediated by PECAM-1: new roles for an old molecule in platelet and vascular cell biology. Arterioscler Thromb Vasc Biol 23(6):953–964. 10.1161/01.ATV.0000071347.69358.D9 12689916 10.1161/01.ATV.0000071347.69358.D9

[CR39] Newman PJ, Berndt MC, Gorski J, White GC, Lyman S, Paddock C, Muller WA (1990) PECAM-1 (CD31) cloning and relation to adhesion molecules of the immunoglobulin gene superfamily. Science 247(4947):1219–1222. 10.1126/science.1690453 1690453 10.1126/science.1690453

[CR40] Newton JP, Hunter AP, Simmons DL, Buckley CD, Harvey DJ (1999) CD31 (PECAM-1) exists as a dimer and is heavily N-glycosylated. Biochem Biophys Res Commun 261(2):283–291. 10.1006/bbrc.1999.1018 10425179 10.1006/bbrc.1999.1018

[CR41] Nualart F, Hein S, Rodríguez EM, Oksche A (1991) Identification and partial characterization of the secretory glycoproteins of the bovine subcommissural organ-Reissner’s fiber complex. Evidence for the existence of two precursor forms. Brain Res Mol Brain Res 11(3–4):227–238. 10.1016/0169-328x(91)90031-r 1661820 10.1016/0169-328x(91)90031-r

[CR42] Nualart F, Cifuentes M, Ramírez E, Martínez F, Barahona MJ, Ferrada L, Saldivia N, Bongarzone ER, Thorens B, Salazar K (2023) Hyperglycemia increases SCO-spondin and Wnt5a secretion into the cerebrospinal fluid to regulate ependymal cell beating and glucose sensing. PLoS Biol 21(9):e3002308. 10.1371/journal.pbio.3002308 37733692 10.1371/journal.pbio.3002308 PMC10513282

[CR43] Pérez J, Garrido O, Cifuentes M, Alonso FJ, Estivill-Torrús G, Eller G, Nualart F, López-Avalos MD, Fernández-Llebrez P, Rodríguez EM (1996) Bovine reissner’s fiber (RF) and the central canal of the spinal cord: an immunocytochemical study using a set of monoclonal antibodies against the RF-glycoproteins. Cell Tissue Res 286(1):33–42. 10.1007/s004410050672 8781210 10.1007/s004410050672

[CR44] Peruzzo B, Pérez J, Fernández-Llebrez P, Pérez-Fígares JM, Rodríguez EM, Oksche A (1990) Ultrastructural immunocytochemistry and lectin histochemistry of the subcommissural organ in the snake natrix Maura with particular emphasis on its vascular and leptomeningeal projections. Histochemistry 93(3):269–277. 10.1007/bf00266388 2312353 10.1007/BF00266388

[CR45] Popescu CP, Hayes H, Meiniel R, Creveaux I, Meiniel A (1997) Short communications: localization of the SCO-spondin gene to cattle chromosome 4. Chromosome Res 5(4):276–277. 10.1023/a:1018479905521 9244457 10.1023/a:1018479905521

[CR46] Reissner E (1860) Beiträge zur Kenntnis vom Bau des Rückenmarks von Petromyzon fluviatilis L. Arch Anat Physiol 77:545–588

[CR47] Rodríguez EM, Oksche A, Hein S, Rodríguez S, Yulis R (1984) Comparative immunocytochemical study of the subcommissural organ. Cell Tissue Res 237(3):427–441. 10.1007/BF00228427 6435876 10.1007/BF00228427

[CR48] Rodríguez S, Rodríguez PA, Banse C, Rodríguez EM, Oksche A (1987) Reissner’s fiber, massa caudalis and ampulla caudalis in the spinal cord of lamprey larvae (Geotria australis): light-microscopic immunocytochemical and lectin-histochemical studies. Cell Tissue Res 247(2):359–366. 10.1007/BF00218317

[CR49] Rodríguez EM, Oksche A, Hein S, Yulis CR (1992) Cell biology of the subcommissural organ. Int Rev Cytol 135:39–121. 10.1016/S0074-7696(08)62038-0 1618609 10.1016/s0074-7696(08)62038-0

[CR50] Rodríguez EM, Rodríguez S, Hein S (1998) The subcommissural organ. Microsc Res Tech 41(2):98–123 9579598 10.1002/(SICI)1097-0029(19980415)41:2<98::AID-JEMT2>3.0.CO;2-M

[CR51] Rodríguez EM, Oksche A, Montecinos H (2001) Human subcommissural organ, with particular emphasis on its secretory activity during the fetal life. Microsc Res Tech 52(5):573–590. 11241867 10.1002/1097-0029(20010301)52:5<573::AID-JEMT1042>3.0.CO;2-6

[CR52] Rose CD, Pompili D, Henke K, Van Gennip JLM, Meyer-Miner A, Rana R, Gobron S, Harris MP, Nitz M, Ciruna B (2020) SCO-spondin defects and neuroinflammation are conserved mechanisms driving spinal deformity across genetic models of idiopathic scoliosis. Curr Biol 30(12):2363-2373e.e6. 10.1016/j.cub.2020.04.020 32386528 10.1016/j.cub.2020.04.020

[CR53] Schoebitz K, Garrido O, Heinrichs M, Speer L, Rodríguez EM (1986) Ontogenetical development of the chick and duck subcommissural organ. An immunocytochemical study. Histochemistry 84(1):31–40. 10.1007/BF00493417 2420757 10.1007/BF00493417

[CR54] Sepúlveda V, Maurelia F, González M, Aguayo J, Caprile T (2021) SCO-spondin, a giant matricellular protein that regulates cerebrospinal fluid activity. Fluids Barriers CNS 18(1):45, 1–24. 10.1186/s12987-021-00277-w 34600566 10.1186/s12987-021-00277-w PMC8487547

[CR55] Simmons DL, Walker C, Power C, Pigott R (1990) Molecular cloning of CD31, a putative intercellular adhesion molecule closely related to carcinoembryonic antigen. J Exp Med 171(6):2147–2152. 10.1084/jem.171.6.2147 2351935 10.1084/jem.171.6.2147 PMC2187965

[CR56] Stanic K, Montecinos H, Caprile T (2010) Subdivisions of chick diencephalic roof plate: implication in the formation of the posterior commissure. Dev Dyn 239(10):2584–2593. 10.1002/dvdy.22387 20730872 10.1002/dvdy.22387

[CR57] Sterba G, Kleim I, Naumann W, Petter H (1981) Immunocytochemical investigation of the subcommissural organ in the rat. Cell Tissue Res 218(3):659–662. 10.1007/bf00210122 7020952 10.1007/BF00210122

[CR58] Sun J, Williams J, Yan HC, Amin KM, Albelda SM, DeLisser HM (1996) Platelet endothelial cell adhesion molecule-1 (PECAM-1) homophilic adhesion is mediated by immunoglobulin-like domains 1 and 2 and depends on the cytoplasmic domain and the level of surface expression. J Biol Chem 271(31):18561–18570. 10.1074/jbc.271.31.18561 8702505 10.1074/jbc.271.31.18561

[CR59] Tian G, Huang L, Xu Z, Lu C, Yuan W, Wu Y, Liao Z, Gao J, Luo Q, Cheng B, Liao X, Lu H (2025) C-mannosyltransferase DPY19L1L-mediated Reissner Fiber formation is critical for zebrafish (*Danio rerio*) body axis straightening. Sci Adv 11(19):eadv2032. 10.1126/sciadv.adv2032 40344050 10.1126/sciadv.adv2032 PMC12063643

[CR60] Tran-Dinh A, Laurent Q, Even G, Tanaka S, Lortat-Jacob B, Castier Y, Mal H, Messika J, Mordant P, Nicoletti A, Montravers P, Caligiuri G, Morilla I (2022) Personalized risk predictor for acute cellular rejection in lung transplant using soluble CD31. Sci Rep 12(1):17628. 10.1038/s41598-022-21070-1 36271122 10.1038/s41598-022-21070-1 PMC9587244

[CR61] Troutwine BR, Gontarz P, Konjikusic MJ, Minowa R, Monstad-Rios A, Sepich DS, Kwon RY, Solnica-Krezel L, Gray RS (2020) The Reissner fiber is highly dynamic in vivo and controls morphogenesis of the spine. Curr Biol 30(12):2353-2362e.e3. 10.1016/j.cub.2020.04.015 32386529 10.1016/j.cub.2020.04.015 PMC7891109

[CR62] Vio K, Rodríguez S, Navarrete EH, Pérez-Fígares JM, Jiménez AJ, Rodríguez EM (2000) Hydrocephalus induced by immunological blockage of the subcommissural organ-Reissner’s fiber (RF) complex by maternal transfer of anti-RF antibodies. Exp Brain Res 135(1):41–52. 10.1007/s002210000474 11104126 10.1007/s002210000474

[CR63] Vio K, Rodríguez S, Yulis CR, Oliver C, Rodríguez EM (2008) The subcommissural organ of the rat secretes Reissner’s fiber glycoproteins and CSF-soluble proteins reaching the internal and external CSF compartments. Cerebrospinal Fluid Res 5(1):3. 10.1186/1743-8454-5-3 18218138 10.1186/1743-8454-5-3 PMC2265671

[CR64] Woollam DHM, Collins P (1980) Reissner’s fibre in the rat: a scanning and transmission electron microscope study. J Anat 131(Pt 1):135–143 7440397 PMC1233292

[CR65] Xu H, Dugué GP, Cantaut-Belarif Y, Lejeune F-X, Gupta S, Wyart C, Lehtinen MK (2023) SCO-spondin knockout mice exhibit small brain ventricles and mild spine deformation. Fluids Barriers CNS 20(1):89. 10.1186/s12987-023-00491-8 38049798 10.1186/s12987-023-00491-8 PMC10696872

[CR66] Yan HC, Baldwin HS, Sun J, Buck CA, Albelda SM, DeLisser HM (1995) Alternative splicing of a specific cytoplasmic exon alters the binding characteristics of murine platelet/endothelial cell adhesion molecule-1 (PECAM-1). J Biol Chem 270(40):23672–80. 10.1074/jbc.270.40.23672 7559536 10.1074/jbc.270.40.23672

[CR67] Zaremba J, Losy J (2002) sPECAM-1 in serum and CSF of acute ischaemic stroke patients. Acta Neurol Scand 106(5):292–298. 10.1034/j.1600-0404.2002.01339.x 12371923 10.1034/j.1600-0404.2002.01339.x

[CR68] Zhang T, Ai D, Wei P, Xu Y, Bi Z, Ma F, Li F, Chen X, Zhang Z, Zou X, Guo Z, Zhao Y, Li J-L, Ye M, Feng Z, Zhang X, Zheng L, Yu J, Li C, Tu T, Zeng H, Lei J, Zhang H, Hong T, Zhang L, Luo B, Li Z, Xing C, Jia C, Li L, Sun W, Ge W (2024) The subcommissural organ regulates brain development via secreted peptides. Nat Neurosci 27(6):1103–1115. 10.1038/s41593-024-01639-x 38741020 10.1038/s41593-024-01639-x

