# Supplementary material for: Anti-PECAM-1 antibodies: another tool for visualization of Reissner´s fiber and the subcommissural organ in rat central nervous system
Source: Brain Struct Funct. 2026 Apr 4;231(3):50. doi: 10.1007/s00429-026-03088-7 (PMC13048961; doi:10.1007/s00429-026-03088-7)
Supplement: Supplementary file 1 — Supplementary Material 1 [file 429_2026_3088_MOESM1_ESM.docx]

**Anti-PECAM-1 antibodies: another tool for visualization of Reissner´s fiber and the subcommissural organ in rat central nervous system**

Lukáš Malčický^1^, Ján Košuth^1^, Daniel Barčák^2^, Jarmila Zrubáková^1^, Marie Vancová^3^, Zuzana Daxnerová^1^, Juraj Ševc^1^, Anna Alexovič Matiašová^1^

*^1^Department of Cell Biology, Institute of Biology and Ecology, Faculty of Science, Pavol Jozef Šafárik University in Košice, Slovak Republic*

*^2^Institute of Parasitology, Slovak Academy of Sciences, Košice, Slovak Republic*

*^3^Laboratory of Electron Microscopy, Institute of Parasitology, Biology Centre, Czech Academy of Sciences, České Budějovice, Czech Republic*

Supplemental Fig. 1


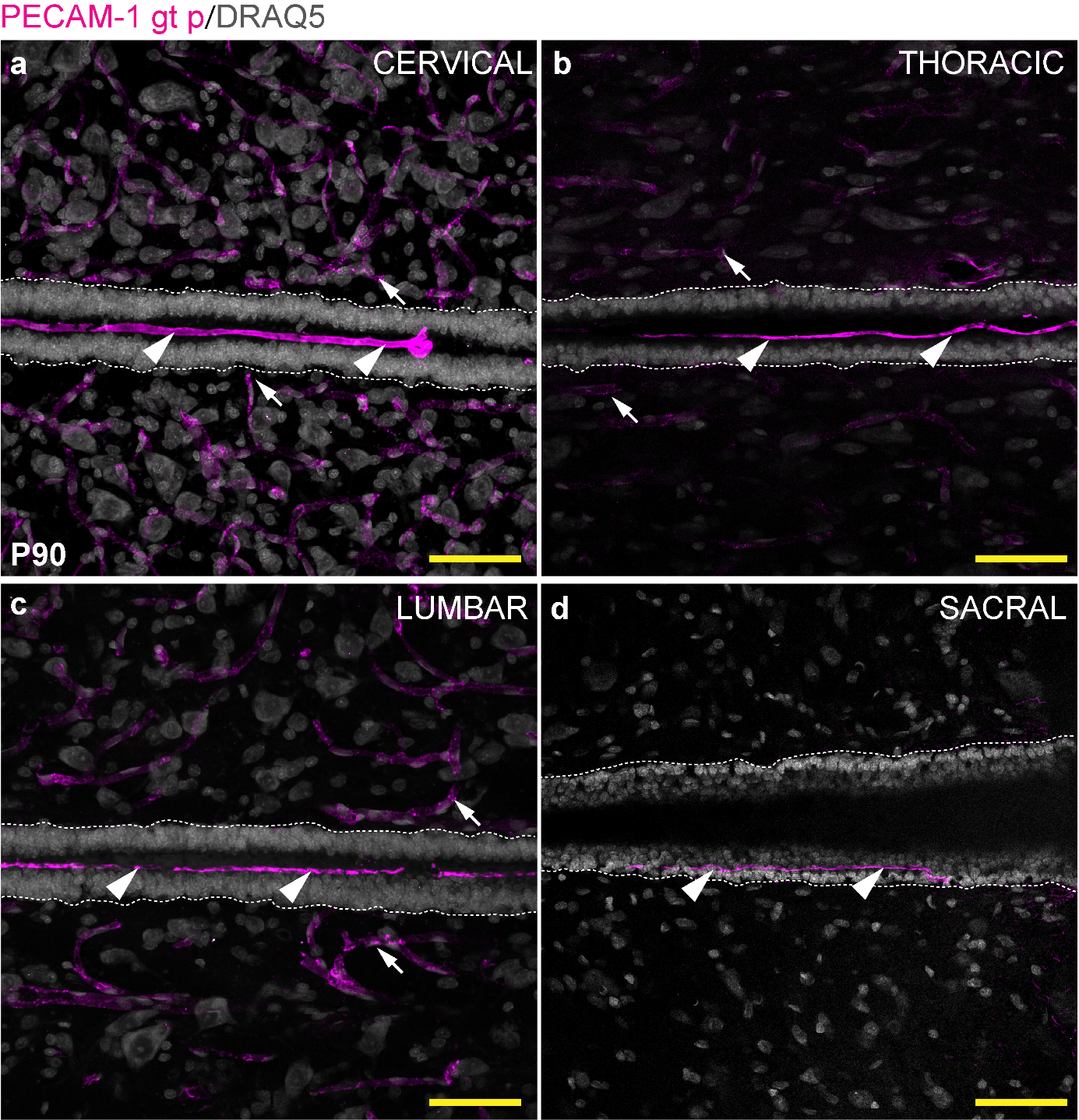


**Supplemental Fig. 1** **Immunoreactivity of blood vessels and RF to goat anti-PECAM-1 antibody in various rat spinal cord regions.** Representative images of cervical (a), thoracic (b), lumbar (c) and sacral (d) spinal cord of rats. PECAM-1^+^ (magenta, goat polyclonal anti-PECAM-1 antibody) parenchymal blood vessels (white arrows) and the elongated structure corresponding to RF (white arrowheads) present in the CC lumen of spinal cord (a-d, Z-stack); the white dashed line in (a-d) corresponds to the CC lining region including the CC lumen and PECAM-1^+^ RF; nuclei of cells (gray, DRAQ5) in (a–d); spinal cord of 90 days old rats (P90); scale bar: a-d = 50 μm.
